# Impact of Macrotexture and Microtexture on the Skid Resistance of Asphalt Pavement Using Three-Dimensional (3D) Reconstruction and Printing Technology

**DOI:** 10.3390/ma18112597

**Published:** 2025-06-02

**Authors:** Fucheng Guo, Jiupeng Zhang, Jianzhong Pei, Haiqi He, Tengfei Yao, Di Wang

**Affiliations:** 1 Key Laboratory of Road & Bridge and Underground Engineering of Gansu Province, Lanzhou Jiaotong University, Lanzhou 730070, China; fcguo@chd.edu.cn (F.G.); tfyao@lzjtu.edu.cn (T.Y.); 2 Key Laboratory of Intelligent Construction and Maintenance of CAAC, Chang’an University, Xi’an 710064, China; 3 Key Laboratory for Special Area Highway Engineering of Ministry of Education, Chang’an University, Xi’an 710064, China; peijianzhong@126.com (J.P.); hehaiqi@chd.edu.cn (H.H.); 4 Department of Civil Engineering, University of Ottawa, 800 King Edward Ave, Ottawa, ON K1N 6N5, Canada

**Keywords:** asphalt pavement, skid resistance, macrotexture, 3D reconstruction, 3D printing technology

## Abstract

In this study, the feasibility of using three-dimensional (3D) printing technology to investigate the impact of macrotexture and microtexture on the skid resistance of asphalt pavement was verified. The macrotexture characteristics of the five types of real asphalt mixtures were captured, reconstructed, and printed. The comparison analysis of the skid resistance between the pavement and printed specimens was conducted, and the correlations and contribution proportions of the macrotexture and microtexture on skid resistance were also calculated. Results show that five printed asphalt mixtures present good consistency in the microtexture with a roughness of about 100 nm. The impact of thin water film on the skid resistance is insignificant for real asphalt mixtures, while it is significant for printed mixtures. The printed specimens under dry conditions show a similar British pendulum number (BPN) with the real pavement specimens under wet conditions, while the BPN under wet conditions for printed specimens are much smaller than the real ones but follows a similar variation trend. Mean profile depth (MPD) values of four printed asphalt concrete (AC) mixtures are well linearly correlated with their BPN under dry and wet conditions, especially for wet conditions with the R^2^ of 0.91. The contribution proportion of macrotexture to the skid resistance is nearly 90% for the dry condition and about 50% for the wet condition.

## 1. Introduction

Researchers have promisingly investigated skid resistance as an important surface performance tightly related to driving safety [1]. Previous studies have shown that skid resistance is influenced by several factors, e.g., vehicle, pavement, and environmental factors [2,3,4]. However, fixed vehicle and environmental conditions are used for conventional measurements; thus, only the pavement factors are addressed in previous investigations [5]. Different types of pavements have been constructed to investigate the effect of pavement on skid resistance, and the results show that the pavement texture plays the dominant role in skid resistance, especially for macrotexture and microtexture [6]. Therefore, the impacts of macrotexture and microtexture on skid resistance have been mainly focused Qualitative results indicated that the macrotexture plays a major role when the vehicle runs at high speed, while the microtexture works dominantly at low driving speeds [7]. Moreover, the macrotexture positively affects the skid resistance when the pavement surface is topped with a water film [8].

Knowing the close link between pavement texture and skid resistance, related investigations have revealed this specific relationship [9,10,11]. The first step for building such a relationship is accurately characterizing the pavement texture. The pavement texture is commonly characterized by the height parameters, e.g., mean texture depth (MTD) and mean profile depth (MPD) [12,13]. MTD is obtained using volumetric methods such as the sand patch method and outflow meter, while MPD is derived from advanced technologies such as laser scanning technology and image processing technology [14,15,16,17]. The correlations are obtained after the pavement texture characterization and skid resistance measurement, while the correlations are unfavorable [18]. The main reason may be that the skid resistance is impacted by both macrotexture and microtexture. However, most of the characterizations of pavement texture focused on the macrotexture, while few parameters can be obtained to characterize the microtexture [19]. Although some attempts were conducted to reveal the impact of macrotexture and microtexture, the quantitative impact and specific contribution of macrotexture and microtexture on skid resistance performance are still unclear for the complex fractal and random characteristics of pavement texture [20].

The conventional asphalt pavement design is a volumetric method involving aggregates of different sizes to ensure good compactness and durability. Thus, the constructed pavement surface shows complex texture characteristics [21]. Such a design method does not lead to accurately defining texture characteristics or exploring the correlations between specific textures and skid resistance. Therefore, the impacts of macrotexture and microtexture on skid resistance should be investigated separately to understand each contribution more clearly. The related investigations should be arranged under simple and controllable conditions [22]. Three-dimensional (3D) printing is a promising technology widely applied to accurately manufacture objects with a special shape and texture [23]. Therefore, this study introduced the technology to investigate the impact of macrotexture and microtexture on skid resistance. According to the theory of Persson, the hysteresis component caused by rough surface texture contact is considered to be the dominating friction mechanism, while the adhesion component caused by the tight contact can be neglected [24]. Therefore, the impact of materials on skid resistance will be ignored in this study.

In this study, the objective is to verify the feasibility of using 3D printing technology to investigate the impact of macrotexture and microtexture on the skid resistance of asphalt pavement. Five kinds of real asphalt mixture specimens were prepared. A laser scanner was used to capture their surface texture characteristics. The pavement specimens with the same macrotexture characteristics were reconstructed using 3D reconstruction technology and manufactured using 3D printing technology. Micro-texture was verified for the printed specimens to eliminate its effect on skid resistance. Finally, the comparison analysis between the real pavement and printed specimens was conducted for the skid resistance under dry and wet conditions. The correlations and contribution proportions of the macrotexture on skid resistance were also calculated. This study presents a method for the 3D reconstruction of real asphalt pavement and a method to separate the contributions of microtexture and macrotexture to the skid resistance, which may be helpful to gaining insight into the formation mechanisms of skid resistance and controlling pavement skid resistance from the perspective of texture.

## 2. Materials and Methods

### 2.1. Preparation of Real Asphalt Pavement Specimens

In this study, SK 90 # base asphalt binder was selected to prepare the asphalt mixture, where 90 # represents that the penetration of binder is within the range of 80 to 100 (unit: 0.1 mm). The basic properties of asphalt binder are shown in Table 1. All properties meet the Chinese technical specification JTG F40-2017 [25]. The coarse and fine aggregate is limestone, and the related properties are shown in Table 2. As for the mineral filler, a limestone filler was selected, and all particle sizes were smaller than 0.075 mm. All their properties satisfy the Chinese technical specification JTG E42-2005 [26].

**Figure 1:**
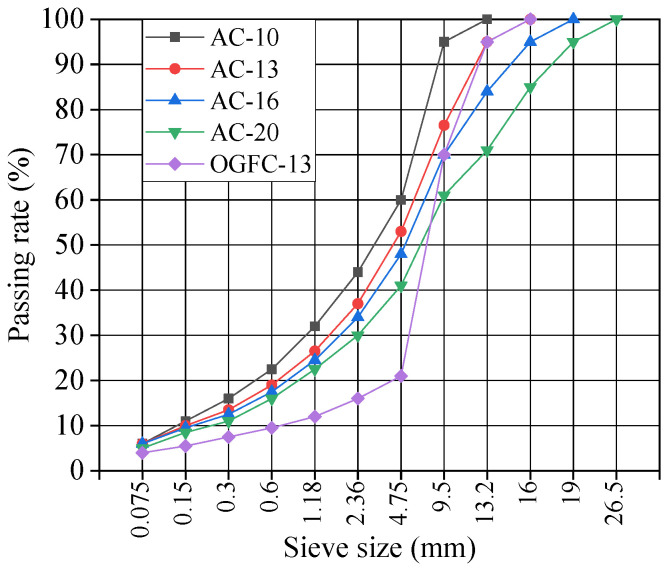
Gradation curves of five asphalt mixtures selected.

After the mixture materials were selected, five kinds of asphalt mixtures were prepared, namely AC-10, AC-13, AC-16, AC-20, and OGFC-13. These five mixtures are commonly used mixtures and have good representatives in asphalt pavement. Different textures were created using different mixture gradations, which was shown in Figure 1. The optimal content of the binder was determined using the Standard Marshall design procedure. The objective of using the optimal binder content is to ensure that the asphalt mix reaches a dense condition and, in addition, has favorable engineering performance.

The Standard Marshall design procedure was used to design the mixtures, and the optimal asphalt binder contents were determined according to the testing procedures [27]. The optimal asphalt binder contents for AC-10, AC-13, AC-16, AC-20, and OGFC-13 are 5.7%, 5.4%, 5.0%, 4.5%, and 5.0%, respectively. The asphalt binder with optimal contents and aggregates with selected gradations was applied to prepare the asphalt mixture slab samples. The loose asphalt mix slabs were compacted with a wheel roller compactor, and the rutting slabs were compacted according to the JTG E20-2011 standard [27]. The size of the compacted rutting slabs was 300 mm × 300 mm × 50 mm. The void content of OGFC-13 is 20%. Three replicate samples were prepared for each mixture.

### 2.2. Capturing of the Surface Macrotexture Topography and Its Optimization

A three-dimensional laser scanning method with high accuracy was adopted in this study to accurately capture the surface macrotexture morphological characteristics of the asphalt mixture surface. The principle of this method is to measure the surface topography of the object at various angles by projecting a highly focused laser beam onto the surface, and the 3D coordinates of each point of the surface topography can be obtained based on the principle of light incidence and reflection. This study adopted a Gocator 2350 series 3D linear laser scanner (provided by Chang’an University, Xi’an city, China) to capture the surface topography of five kinds of asphalt pavement. The scanning speed, scanning frequency, *x*-axis, and *z*-axis resolution were set as 100 mm/s, 400 Hz, 0.25 mm, and 0.041 mm, respectively. The elevation coordinates for each point on the specimens’ surface were captured with intervals of 0.25 mm in the x and y coordinate directions.

**Figure 2:**
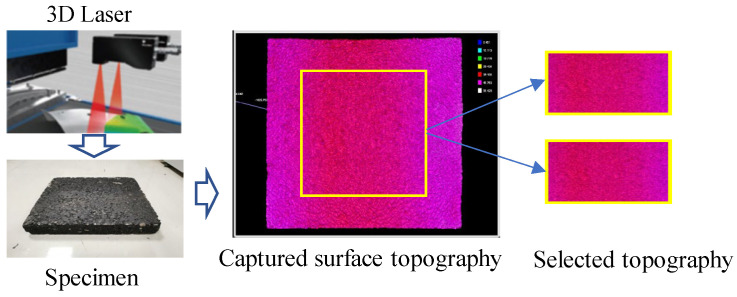
Capturing process of surface topography for five asphalt mixtures selected.

The 3D coordinates of the specimens’ surface topography can be obtained using the scanner. However, some data around the boundary is missed because the capturing process is prone to being disturbed by the specimens’ construction processing and environmental factors. Therefore, the captured data should be refined by selecting only the most representative scanned area (within the yellow box). The boundary data was deleted, and the surface topography around the central point with an area of 200 mm × 200 mm was selected. The capturing process of surface topography can be summarized in Figure 2.

### 2.3. 3D Reconstruction and 3D Printing of Specimens

**Figure 3:**
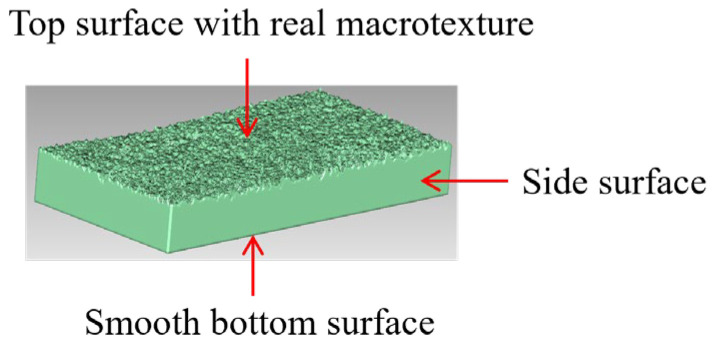
Reconstructed mixture specimen topped with the reconstructed pavement macrotexture.

The 3D reconstruction should be conducted after the surface topography of the pavement specimens is collected and filtered. The focus of this study is to investigate the impact of pavement surface texture on skid resistance; thus, only the top surface topography of the specimens was captured by the scanner, which can eliminate the interference of other irregular real surfaces on the results and reduce the acquisition cost as well. However, the obtained top surface data cannot be directly used for the 3D reconstruction and printed as a specimen for the surface data missing from other surfaces. It is necessary to form a complete hexahedron structure for printing. Therefore, a hexahedral 3D structure with a smooth bottom, four side surfaces, and real surfaces with pavement macrotexture was designed with the size of 200 mm × 100 mm in *x* and *y* directions, as shown in Figure 3.

The 3D reconstruction procedures include (1) Converting the elevation coordinates data of the surface by laser scanner into three-dimensional coordinate format (*x*, *y*, *z*) and saving them in the TXT document, where the interval in x and y coordinate directions is 0.25 mm; (2) Write the coordinates generation code of the bottom and four side surfaces using Visual Studio Code 2023 software, and run the code using the Python 3.7 software to generate the coordinates data and save them as another different TXT document; (3) Merge all coordinates together to save them as one TXT document, and import the document into Geomagic Studio 2013 software to obtain the point cloud pavement structure with the real macrotexture of the asphalt mixtures; (4) Optimize the surface point cloud data and encapsulate the optimized point cloud to form a polygon structure, and fully optimize the polygon structure through multiple polygon optimization programs to eliminate the unfavorable mesh structure, and finally export the optimized polygon structure in the form of stereolithography (STL) files.

**Figure 4:**
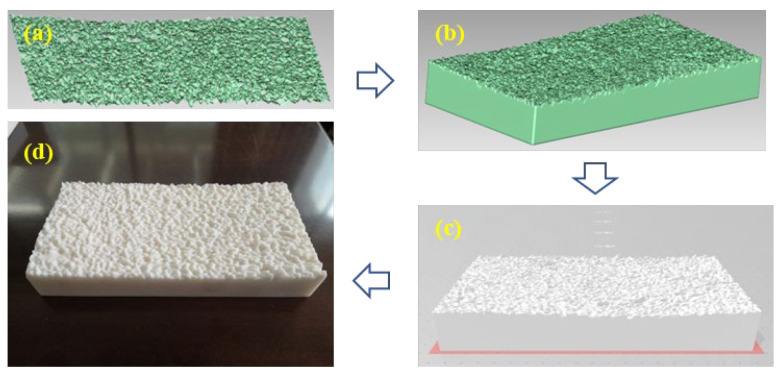
3D reconstruction and printing process: (**a**) Captured surface topography, (**b**) Point cloud of pavement structure, (**c**) Polygon structure of STL form, and (**d**) Printed specimen.

The reconstructed specimens with STL printable files were fed into the 3D printer to print the specimens, where a Lite600HD 3D printer (provided by Shenzhen Wenext Technology Co., Ltd., Shenzhen, China) was used. The maximum printable size of this printer is 600 mm × 600 mm × 400 mm, and its printing accuracy is 0.2 mm. The Future R4600 resin material was selected as the printing material; this is an acrylonitrile-butadiene-styrene (ABS) copolymer-like stereolithography resin material with precise forming processability and durability properties. Moreover, this resin material was widely used as the 3D printing material to print the components with the high precision and toughness requirements, and the printed models will present good performance with the stable model size, low shrinkage rate, excellent yellowing resistance, and excellent processability. The specimens with the same macrotexture can be printed using the 3D reconstruction procedures and 3D printer. The 3D reconstruction and printing process can be summarized in Figure 4.

### 2.4. Microtexture Measurement of the Printed Specimens

This study aimed to investigate the influence of macrotexture; thus, the effect of microtexture should be eliminated. Only macrotexture was captured using intervals of 0.25 mm. These printed specimens must have a similar microtexture on any of their flat surfaces because the variation of the microtexture only depends on the printing process formed by the layer-by-layer overlapping of the materials. In this study, the microtexture verification was conducted on the smooth bottom surface by atomic force microscopy (AFM) testing and skid resistance measurement to ensure that the bottom surface has similar microtexture and skid resistance performance [28].

An AFM from Bruker Dimension (Karlsruhe, Germany) was used to measure the microtexture topography, where the tapping mode was applied, selecting a measurement area of 30 μm × 30 μm. The microtexture of the smooth bottom surface was captured and characterized using the roughness parameters of the arithmetic mean deviation (*R_a_*) and the root mean square (*R_q_*). The corresponding calculation equations can be shown in Equations (1) and (2).

(1) $$R_{a}=\frac{\int_{0}^{l}\left|Z(x)\right|dx}{l}=\frac{1}{n}\sum_{i=1}^{n}\left|Z_{i}\right|,$$

(2) $$R_{q}=\sqrt{\frac{\int_{0}^{l}\left|Z(x)\right|dx}{l}}=\sqrt{\frac{1}{n}\sum_{i=1}^{n}\left|Z_{i}\right|},$$

where *Z*(*x*) and *Z_i_* are the ordinate values in the profile curve, *l* is the evaluation length, and *n* is the number of the scattered points within *l*.

**Figure 5:**
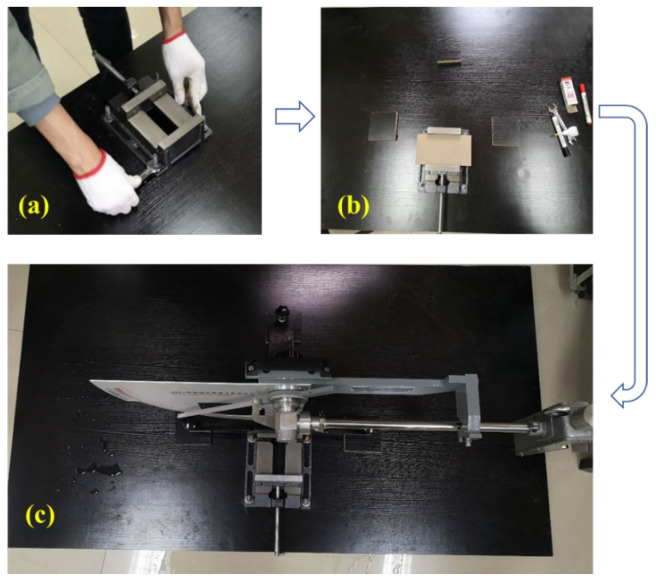
Specimen fixing platform for the BPN measurement: (**a**) Install the parallel-jaw vice on a flat multilayer board; (**b**) Fix the specimen; and (**c**) Measure the BPN [30].

Moreover, the skid resistance of the bottom surface was also measured for further verification of the microtexture. The skid resistance was characterized by the British pendulum number (BPN) using a British pendulum friction tester in this study [29]. The British pendulum tester (BPT) has been widely used in previous works to measure skid resistance with good reliability [29]. Unlike the conventional measurement, the verifications were conducted using printed specimens with a size of nearly 200 mm × 100 mm × 25 mm. Although such small specimens meet BPT measurement requirements, they are hard to measure due to their weight and tendency to move easily. Therefore, a specimen-fixing platform was designed to fix the printed specimen during the skid resistance measurement to ensure its reliability. The assembly process of the specimen-fixing platform is shown in Figure 5 [30]. The testing temperature is set as 20 °C. During the BPT testing for the bottom surfaces, the specimen was inverted to ensure the bottom surface was upward and horizontal.

### 2.5. Comparison of Skid Resistance of Real and Printed Specimens

**Figure 6:**
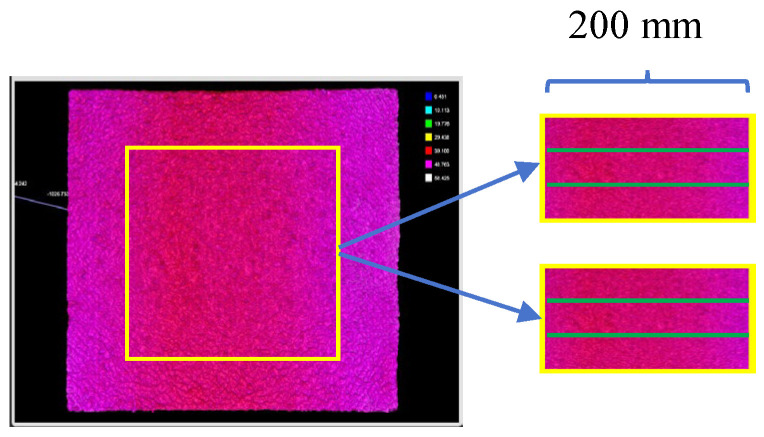
Selected point cloud data for the MPD value calculation (green line).

The skid resistance of the real asphalt mixture specimens was indirectly characterized by mean texture depth (MTD) values. The MPD values were calculated using the captured surface texture coordinates for each mixture specimen, where four-point cloud data lines (as shown in Figure 6) were selected and halved to calculate MPD values for each mixture specimen (because the MPD value was calculated with the reference line of 100 mm), and the final averaged MPD values were obtained using eight calculated MPD values.

Moreover, the skid resistance of the asphalt mixture specimens was directly measured using BPT, while the skid resistance of the printed asphalt mixture specimens was measured using BPT and the specimens fixing platform. The BPN values were obtained for each specimen under dry and wet conditions for the full comparison. The comparison of BPN was conducted to verify the feasibility of using 3D printing technology to investigate the skid resistance of asphalt pavement. Moreover, the impact of macrotexture on skid resistance was analyzed using linear regression between MPD and BPN for five printed mixture specimens. The contribution of pavement macrotexture to the skid resistance was also analyzed.

## 3. Results and Discussion

### 3.1. Microtexture Verification of the Printed Specimens

**Figure 7:**
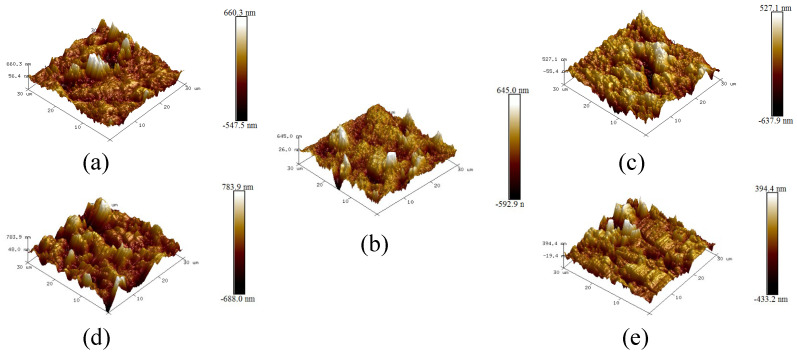
3D topography of the smooth bottom surfaces for five printed specimens: (**a**) AC-10, (**b**) AC-13, (**c**) AC-16, (**d**) AC-20, and (**e**) OFGC-13.

The microtexture of the smooth bottom surface was measured using AFM, and the 3D topography of the smooth bottom surface for five printed specimens is shown in Figure 7.

**Figure 8:**
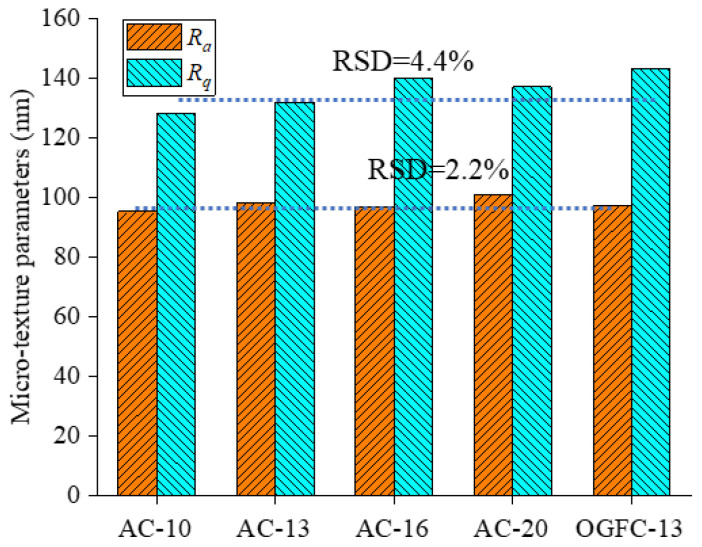
Microtexture parameters of the smooth bottom surfaces for five printed specimens.

As shown in Figure 7, the variation range of the 3D topography in the *z*-direction for bottom surfaces is within 1500 nm, which means that the printed specimens have a small and similar microtexture compared to the real asphalt pavement. Moreover, the surface height variation shows a similar range for five specimens, which means good stability in structure manufacturing. This may be because the same printing table was used and the printing conditions are kept constant for all printed samples, resulting in the same topography of the bottom of the samples. The microtexture roughness parameters *R_a_* and *R_q_* are calculated according to the obtained 3D topography characteristics for verifying the microtexture characteristics quantitatively. The variations of *R_a_* and *R_q_* values are shown in Figure 8. The average line and the relative standard deviation (RSD) are also depicted.

As shown in Figure 8, the *R_a_* values of the smooth bottom surfaces for five printed specimens vary from 95.2 to 101, while the *R_q_* values vary from 128 to 143. The RSD values of *R_a_* and *R_q_* are 2.2% and 4.4%, respectively. It means that the smooth bottom surfaces present a similar microtexture with small roughness values and roughness variations. Therefore, five printed specimens show good consistency in the microtexture characteristics from the surface topography results.

**Figure 9:**
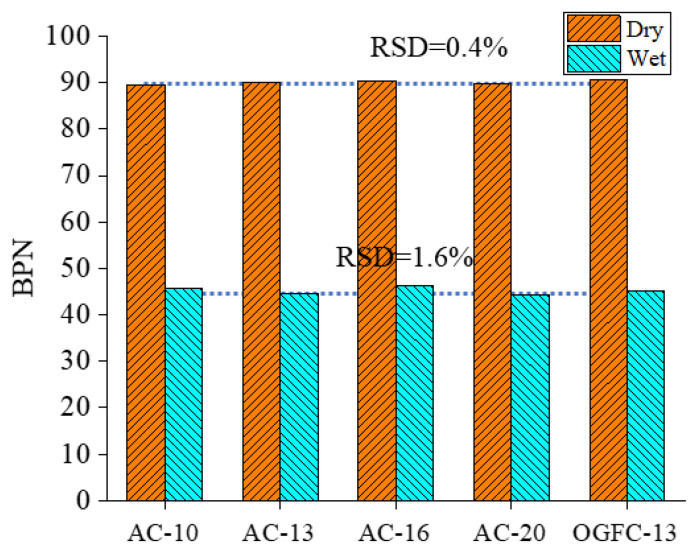
BPN values of the smooth bottom surfaces for five printed specimens.

To further verify the similarity of the microtexture characteristics, the skid resistance was also measured for the smooth bottom surfaces of five specimens. The BPN results are measured under dry and wet conditions, as shown in Figure 9.

As shown in Figure 9, the BPN of the smooth bottom surfaces varies from 89.9 to 90.5 under dry conditions, while it varies from 44.4 to 46.2 under wet conditions. The RSD values of BPN are 0.4% and 1.6% for the dry and wet conditions, respectively. The BPN of the smooth bottom surfaces shows similar values for five printed specimens with minimal variations. Therefore, it can be concluded that the five printed specimens have good consistency in the microtexture based on the microtexture parameters using AFM tests and BPN values using BPT testing, and the good printing accuracy and stability are also verified. Thus, the impact of microtexture on the skid resistance can be eliminated for good consistency when investigating the macrotexture effect.

### 3.2. Comparisons of Skid Resistance for Real and Printed Specimens

**Figure 10:**
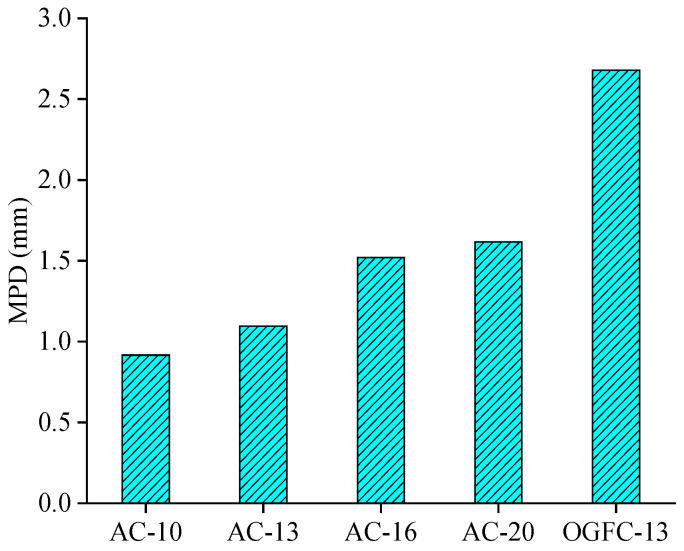
MPD values for five different asphalt mixtures.

The skid resistance of five different asphalt mixtures was indirectly characterized using MPD values, and the corresponding results are shown in Figure 10.

As shown in Figure 10, the MPD values show the distinguished difference for different mixtures, which means that the selected mixtures represent well the different characteristics of the macrotexture. The MPD values increase with the nominal maximum aggregate size for AC mixtures; namely, they show the increased skid resistance. Moreover, the MPD value for the OGFC-13 mixture is considerably larger than those for the AC mixtures, which means that the former mixture shows greater skid resistance. This conclusion is consistent with the previous studies, which showed that the OGFC had the highest macrotexture depth (about 2.5 mm) [31]. The MPD value is just a description from the texture side, and the skid resistance should be further evaluated and compared using the direct evaluation index.

**Figure 11:**
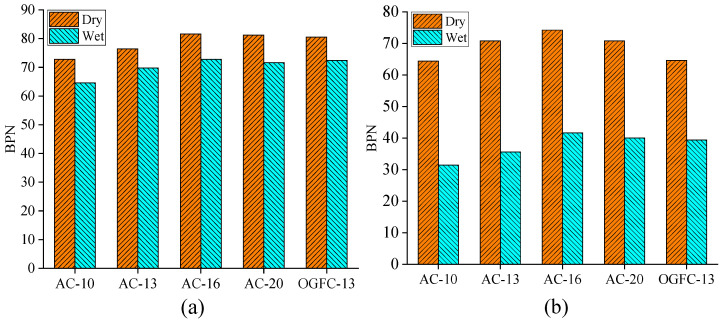
BPN results for five real mixture and printed specimens: (**a**) real mixtures; (**b**) printed specimens.

Furthermore, the skid resistance of real asphalt mixture specimens and printed specimens was directly evaluated using BPT, and the BPN results for the five real mixture and printed specimens under dry and wet conditions are shown in Figure 11.

As shown in Figure 11a, the variations of the BPN values under the dry and wet conditions for five kinds of real asphalt mixture specimens are insignificant with the change of the mixture gradations, and its variations are within 10. For AC types of asphalt mixtures, the BPN values increase and decrease with the nominal maximum aggregate size. This may be because the increase in aggregate size will induce the large energy dissipation when the rubber slider slides on the pavement, increasing the skid resistance. However, an excessive aggregate size will reduce the contact area throughout the whole sliding process, which may reduce the time of energy dissipation and further decrease the skid resistance. Compared to AC mixtures, the BPN of the OGFC mixture is slightly greater than that of the AC mixture under the same nominal maximum nominal aggregate size, which is similar to previous works. Therefore, a good mixture should be selected for good skid resistance.

The impact of water film on the skid resistance for the real asphalt mixtures is insignificant, which may be caused by the low-speed (about 10 km/h) measurement of skid resistance. The slider rubber can penetrate the water film and directly contact the pavement’s top texture. Moreover, it should be noted that the BPN values obtained in this study are slightly smaller than the results from the previous publications [32], which may be because the skid resistance measurement was carried out on the specimens with the new molding, while the measurements are often measured on the surface with the short-term service, where the coated asphalt binder on aggregates will be partially removed.

As shown in Figure 11b, the skid resistance of the printed specimens is considerably reduced when the surface condition changes from dry to wet. The reason can be attributed to the small microtexture characteristics with the *R_a_* values of about 100 nm for the printed specimens; thus, most of the microtexture “valley” on the surface may be sealed by the water, and its contributions to skid resistance will be considerably impaired [33]. Compared to Figure 11a,b, the printed specimens under dry conditions show a similar BPN to the real pavement specimens under wet conditions, while the BPN for printed specimens under wet conditions is much smaller than that of the real specimens. However, the skid resistance of five printed specimens under the dry and wet conditions shows a similar variation to that of the real mixture specimens. Namely, it increases firstl and decreases afterward with the aggregate size. Therefore, it can be concluded that the variations of skid resistance with the aggregate size are derived from the macrotexture, while the contribution of the microtexture is similar.

### 3.3. Impact of Macrotexture on Skid Resistance

**Figure 12:**
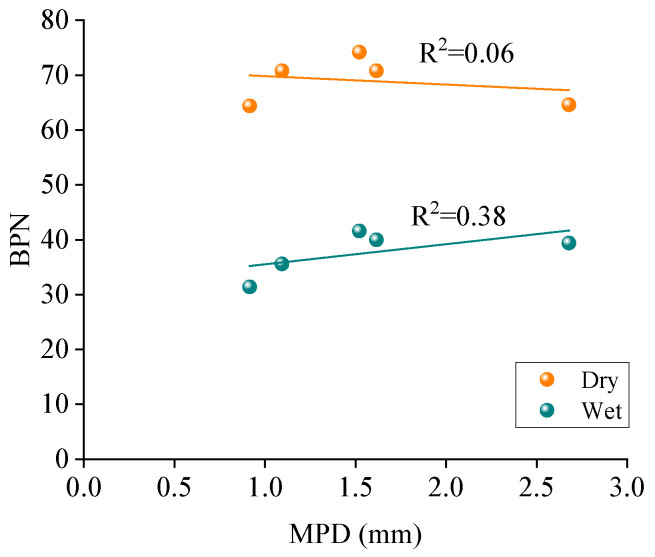
Correlation of MPD and BPN for five printed specimens.

The impact of macrotexture on skid resistance was analyzed using the linear regression between MPD and BPN for five printed specimens, as shown in Figure 12.

As shown in Figure 12, there is no linear correlation between MPD and BPN for five printed specimens under dry conditions, where the coefficient of determination (R^2^) is equal to 0.06. The linear correlation between MPD and BPN under wet conditions is weak, where the coefficient of determination (R^2^) is equal to 0.38. This means that the MPD value is insufficient to fully characterize the skid resistance performance for different types of asphalt pavement. The skid resistance cannot be effectively improved by simply increasing the MPD values. The printed OGFC mixture specimen has an extremely large MPD value of 2.68 mm, while its BPN is only 39.4. Several publications have shown that the BPN values are not well linearly correlated with the MPD values for different mixtures [30,34].

**Figure 13:**
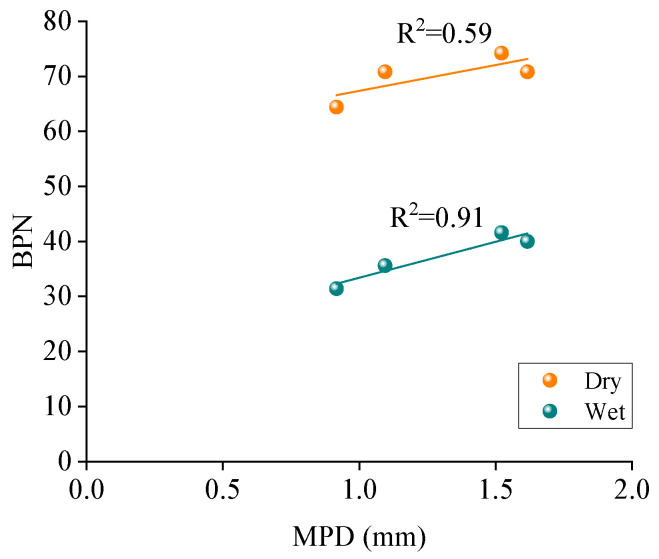
Correlation of MPD and BPN for four printed AC-mixture specimens.

The correlation of MPD and BPN for five printed specimens may be considerably influenced by the OGFC mixture due to its large MPD value. The correlation of MPD and BPN was further analyzed for the AC mixtures, as shown in Figure 13.

As shown in Figure 13, MPD values are well linearly correlated with their BPN under dry and wet conditions, especially for wet conditions with an R^2^ of 0.91. It shows that the AC mixture specimens with macrotexture have good skid resistance with large MPD values. The reason might be that the same AC mixtures will show similar surface texture shape and structure characteristics, and the large energy dissipation will occur when the rubber slides on the specimens with large MPD values. However, the considerably large MPD is not always effective since tires only partially envelop the top texture of the pavement surface [35].

**Figure 14:**
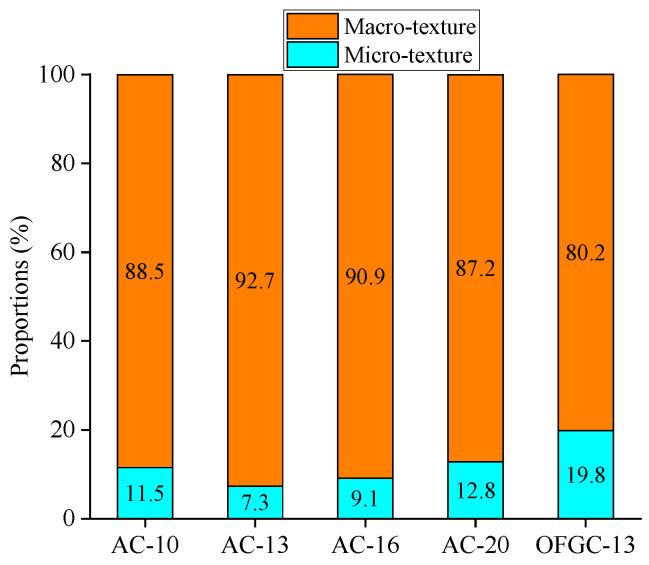
Contribution proportions of the macrotexture and microtexture to skid resistance under dry conditions.

**Figure 15:**
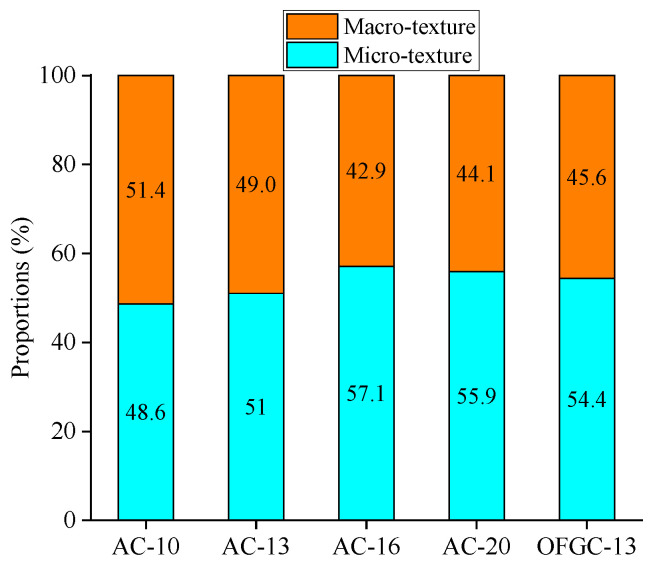
Contribution proportions of the macrotexture and microtexture on skid resistance under wet conditions.

For further analyzing the contribution of macrotexture to the skid resistance, the BPN value is separated into two sections. One of them is derived from macrotexture, while the other is derived from microtexture. The hypothesis of the impact of the materials and optimization variations on the skid resistance is ignored in this calculation. The calculated contribution proportions of macrotexture and microtexture to skid resistance are shown in Figure 14 and Figure 15.

As shown in Figure 14 and Figure 15, the variations of the contribution proportions of the macrotexture and microtexture on skid resistance are insignificant with the mixture type, and it shares a similar trend with the variation of BPN. However, the contribution proportions show a remarkable difference when the pavement surface conditions change from dry to wet. The proportion of macrotexture on the skid resistance is nearly 90% in all skid resistance components for the dry condition, while it is about 50% for the wet condition. This means that macrotexture plays a dominant role in dry conditions, while microtexture and macrotexture work in wet conditions when the measurement speed is low.

The skid resistance is relatively high for both real and printed specimens under dry conditions; therefore, the impact of microtexture is insignificant. The microtexture is sealed with water under wet conditions, resulting in relatively large variations between real and printed specimens. Moreover, the proportions may be because the contribution of the macrotexture to skid resistance is overestimated under the dry condition, while the contribution is underestimated under the wet condition. The microtexture with the Nanoscale roughness still works when the surface is dry. The macrotexture may be affected during the reconstruction process. Therefore, this proportion needs to be further revised in future works.

## 4. Conclusions

In this study, the feasibility of using 3D printing technology to investigate the impact of macrotexture on the skid resistance of asphalt pavement was verified. The macrotexture characteristics of the real asphalt mixture specimens were captured, reconstructed, and printed. The comparison analysis of the skid resistance between the real pavement and printed specimens was conducted, and the correlations and contribution proportions of the macrotexture and microtexture on skid resistance were also calculated. The following conclusions can be obtained.

(1) 3D reconstruction methods and 3D printing technology are feasible to achieve the reproduction of the macrotexture of asphalt pavement. The results from AFM show that five printed specimens have similar microtexture, where the RSD values of *R_a_* and *R_q_* are 2.2% and 4.4%, respectively. The BPN of the smooth bottom surfaces was also measured; the RSD values of BPN are 0.4% and 1.6% for the dry and wet conditions, respectively. It can be concluded that the printed specimens have similar microtexture, and the effect of microtexture on skid resistance can be ignored for the further analysis is of macrotexture effect.
(2) The variations of the BPN values under the dry and wet conditions for five kinds of real asphalt mixture specimens are insignificant with the change of the mixture type, and its variations are within 10 units. The impact of thin water film on the skid resistance for the real asphalt mixtures is insignificant, while the skid resistance of the printed specimens is considerably reduced when the surface condition changes from dry to wet. The printed specimens under dry conditions show a similar BPN to the real pavement specimens under wet conditions, whereas, although the BPN for specimens printed in wet conditions are much smaller than for real specimens, they follow a similar variation trend.
(3) MPD values of four printed AC mixtures are well linearly correlated with their BPN for AC mixtures under dry and wet conditions, especially for wet conditions with R^2^ of 0.91. The variations of the macrotexture and microtexture contribution proportions on skid resistance are insignificant with the mixture type. The contribution proportions show a remarkable difference when the pavement surface conditions change from dry to wet. The proportion of macrotexture on the skid resistance is nearly 90% for the dry conditions and about 50% for the wet conditions.

This study provides a new method to investigate the impact of macrotexture on skid resistance using 3D printing technology. The limitations of the study are the limited mixtures and materials that were used. Therefore, the different mixtures can be further investigated, and the different texture ranges and parameters can be further extracted using 3D technology in future works. In addition, different printing materials will also be selected.

## Figures and Tables

**Table 1:** Basic properties of asphalt binder.

Properties	Measured Values	Standard Values
Specific gravity (g/cm^3^)	1.19	-
Penetration (0.1 mm, 25 °C)	90.5	80~100
Ductility (cm, 15 °C)	136	≥100
Softening point (°C)	46.5	≥44

**Table 2:** Basic properties of coarse and fine aggregates.

Aggregate Types	Properties	Aggregate Sizes (mm)					Standard
		19 mm	16 mm	13.2 mm	9.5 mm	4.75 mm	
Coarse	Specific gravity (g/cm^3^)	2.723	2.731	2.746	2.749	2.762	≥2.5
	Water absorption (%)	1.23	1.08	1.03	0.86	0.59	≤3.0
	Crashing value (%)	11.3					≤25
	Polished stone value	45					≥40
	Elongated particle contents (%)	8.9					≤18
Fine	Specific gravity (g/cm^3^)	2.782					≥2.5
	Sand equivalent (%)	68					≥60
	methylene blue	20					≤25
	<0.075 mm Particles content (%)	1.65					≤3

## Data Availability

The original contributions presented in the study are included in the article; further inquiries can be directed to the corresponding author.
